# Metformin and growth differentiation factor 15 (GDF15) in type 2 diabetes mellitus: A hidden treasure

**DOI:** 10.1111/1753-0407.13334

**Published:** 2022-11-28

**Authors:** Hayder M. Al‐kuraishy, Ali I. Al‐Gareeb, Athanasios Alexiou, Marios Papadakis, Eman Hassan Nadwa, Sarah M. Albogami, Mohammed Alorabi, Hebatallah M. Saad, Gaber El‐Saber Batiha

**Affiliations:** ^1^ Department of Clinical Pharmacology and Medicine, College of Medicine AL‐Mustansiriyah University Baghdad Iraq; ^2^ Department of Science and Engineering Novel Global Community Educational Foundation Hebersham Australia; ^3^ AFNP Med Wien Austria; ^4^ Department of Surgery II, University Hospital Witten‐Herdecke, Heusnerstrasse 40 Wuppertal Germany; ^5^ Department of Pharmacology and Therapeutics College of Medicine, Jouf University Sakakah Saudi Arabia; ^6^ Department of Medical Pharmacology, Faculty of Medicine Cairo University Giza Egypt; ^7^ Department of Biotechnology College of Science, Taif University Taif Saudi Arabia; ^8^ Department of Pathology, Faculty of Veterinary Medicine Matrouh University Marsa Matruh Egypt; ^9^ Department of Pharmacology and Therapeutics, Faculty of Veterinary Medicine Damanhour University Damanhour Egypt

**Keywords:** growth differentiation factor 15 (GDF15), metformin, type 2 diabetes mellitus, 2型糖尿病, 二甲双胍, 生长分化因子15

## Abstract

Type 2 diabetes mellitus (T2DM) is a chronic endocrine disorder due to the reduction of insulin sensitivity and relative deficiency of insulin secretion. Growth differentiation factor 15 (GDF15) belongs to the transforming growth factor beta (TGF‐β) superfamily and was initially identified as macrophage inhibitory cytokine‐1 (MIC‐1). GDF15 is considered a cytokine with an anti‐inflammatory effect and increases insulin sensitivity, reduces body weight, and improves clinical outcomes in diabetic patients. GDF15 acts through stimulation of glial‐derived neurotrophic factor (GDNF) family receptor α‐like (GFRAL), which is highly expressed in the brain stem to induce taste aversion. Metformin belongs to the group of biguanides that are derived from the plant *Galega officinalis*. It is interesting to note that metformin is an insulin‐sensitizing agent used as a first‐line therapy for T2DM that has been shown to increase the circulating level of GDF15. Thus, the present review aims to determine the critical association of the GDF15 biomarker in T2DM and how metformin agents affect it. This review illustrates that metformin activates GDF15 expression, which reduces appetite and leads to weight loss in both diabetic and nondiabetic patients. However, the present review cannot give a conclusion in this regard. Therefore, experimental, preclinical, and clinical studies are warranted to confirm the potential role of GDF15 in T2DM patients.

## Graphical abstract

**Highlights**

- Metformin activates GDF15 expression, which reduces appetite with induction of weight loss in both diabetic and non‐diabetic patients.
- GDF15 level is linked and correlated with the progression of diabetic complications including thrombosis, diabetic nephropathy, diabetic neuropathy, and diabetic retinopathy.

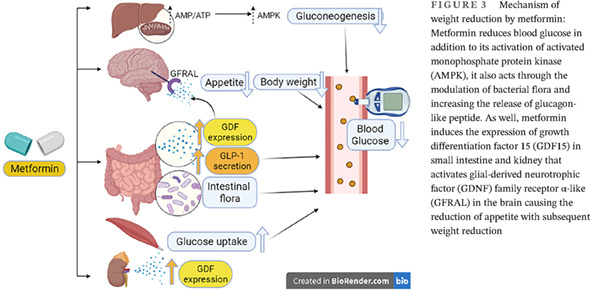

## 摘要

2型糖尿病(T2DM)是一种因胰岛素敏感性降低和胰岛素分泌相对不足而引起的慢性内分泌疾病。生长分化因子15 (GDF15)属于转化生长因子‐β (TGF‐β)超家族，最初被鉴定为巨噬细胞抑制因子‐1 (MIC)。GDF15被认为是一种具有抗炎作用、增加胰岛素敏感性、减轻体重和改善糖尿病患者临床疗效的细胞因子。GDF15通过刺激脑干高表达的胶质源性神经营养因子(GDNF)家族受体α样(GFRAL)诱导味觉厌恶。二甲双胍属于从大戟属植物中提取的双胍类化合物。二甲双胍是一种胰岛素增敏剂，用于T2DM的一线治疗，已被证明可增加GDF15的循环水平。本综述旨在确定GDF15生物标志物在T2DM中的关键作用以及二甲双胍药物是如何影响它发。综述表明，二甲双胍激活GDF15的表达，降低糖尿病和非糖尿病患者的食欲，诱导体重减轻。然而，目前的研究无法在这方面给出结论。因此，有必要进行实验、临床前和临床研究来确认GDF15在T2DM患者中的潜在作用。

## 1 INTRODUCTION

Growth differentiation factor 15 (GDF15) was initially identified as macrophage inhibitory cytokine‐1 (MIC‐1). GDF15 belongs to the transforming growth factor beta (TGF‐β) superfamily and is regarded as a stress response member of TGF‐β.^1^ It is usually found at a low concentration, except in the placenta, so GDF15 is increased during pregnancy and following organ injury, especially in the lungs and liver.^1^ The clear function of GDF15 is still not well recognized, though it plays a crucial role in the regulation of cell growth, apoptosis, and inflammatory activation.^2^ Therefore, GDF15 is regarded as a prognostic biomarker in cancer, inflammatory diseases, and cardiovascular complications.^1^, ^2^ GDF15 may act as anti‐inflammatory and pro‐inflammatory signaling in different cardiovascular complications. It has been shown that the p53 protein promotes the expression of GDF15 during inflammation and oxidative stress.^3^, ^4^, ^5^ The release of GDF15 is stimulated by various growth factors and cytokines including TGF‐β, tumor necrosis factor alpha (TNF‐α), interleukin‐1β (IL‐1β), macrophage colony‐stimulating factor (M‐CSF), angiotensin II, and p53.^6^, ^7^

**FIGURE 1:**
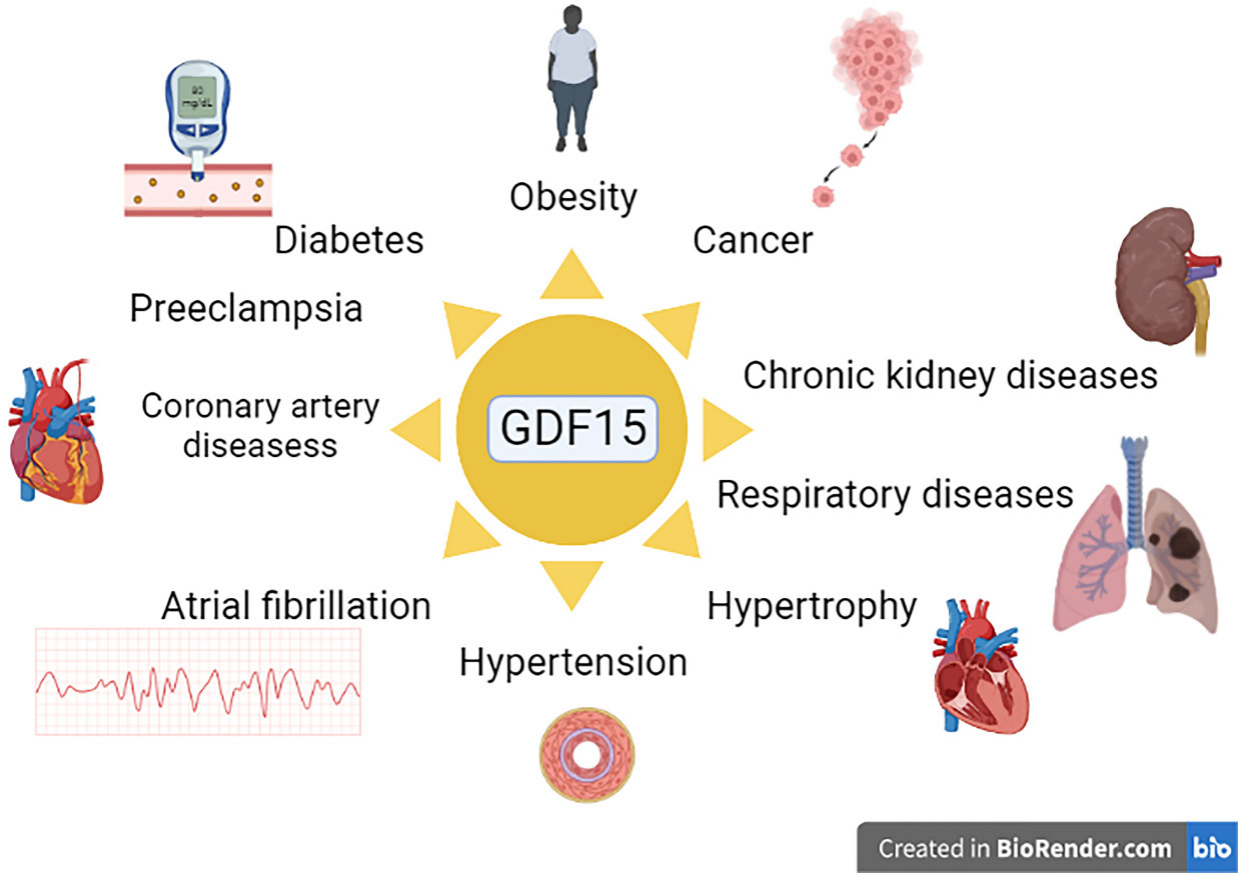
Role of growth differentiation factor 15 (GDF15) in different disorders

GDF15 was implicated in different cardiometabolic disorders and cancer.^8^ However, recent studies observed that GDF15 is regarded as a cytokine that has an anti‐inflammatory effect and increases insulin sensitivity, which may reduce body weight and improve clinical outcomes in diabetic patients.^8^ In healthy subjects, the normal expression of GDF15 reduces appetite and inflammation with the improvement of insulin sensitivity.^3^ GDF15 induces weight loss by suppressing appetite, so neutralizing the antibodies against GDF15 reduces cancer‐induced cachexia in mice.^3^ Though, in chronic metabolic and inflammatory disorders, the overexpression of GDF15 induces desensitization of central and peripheral receptors of GDF15 with subsequent elevation of GDF15 serum levels.^8^, ^9^ Thus, GDF15 is implicated in the pathology of different disorders (Figure 1).

It is interesting to note that metformin, which is an insulin‐sensitizing agent used as a first‐line treatment of type 2 diabetes mellitus (T2DM), has been shown to increase the circulating level of GDF15.^10^, ^79^

Therefore, the objective of the present review is to determine the potential role of GDF15 in T2DM and how metformin affects it.

### 1.1 T2DM and GDF15

T2DM is a chronic endocrine disorder due to the reduction of insulin sensitivity and relative deficiency of insulin secretion.^11^ The development of T2DM is closely linked with overweight, obesity, and increased age.^11^, ^80^ Impaired insulin secretion and insulin resistance (IR) are still the core defect in T2DM that is linked to the development of macrovascular and microvascular complications.^12^, ^13^ Many pro‐inflammatory/inflammatory cytokines and associated mediators are released from the visceral to control pancreatic β‐cell functions. For example, TNF‐α, plasminogen activator inhibitor 1 (PAI‐1), and different adipocytokines are increased in T2DM and associated complications.^6^, ^75^ One of the most important metabolic regulators is GDF15, which acts like leptin and adiponectin and is therefore called a cardiokine.^14^ Like other adipokines, GDF15 reduces body weight and visceral adiposity by decreasing appetite and food intake.^14^ Many human studies observed that GDF15 serum level was correlated with blood glucose, adiposity, and body mass index. For example, GDF15 serum level was 275–411 ng/ml in healthy subjects and increased to 344–626 ng/ml in obese patients.^15^ A cohort study including 118 obese patients and 30 healthy controls showed that GDF15 serum level was higher in obese patients compared to controls.^15^ Besides, GDF15 serum level is positively correlated with age, waist–hip ratio, blood pressure, blood glucose, glycosylated hemoglobin (HbA1c), C‐peptide, and homeostatic model assessment of insulin resistance (HOMA‐IR).^15^ Of note, IR, old age, and creatinine serum levels are regarded as independent predictors for high GDF15 serum levels.^15^ Thus, GDF15 was proposed to be of therapeutic value in the management of IR, T2DM, and obesity through the modulation of metabolic activity of the lipolytic genes.^16^ In addition, GDF15 increases lipolysis, thermogenesis, and metabolism of oxidative metabolites, thereby reducing the risk of developing obesity, IR, and related oxidative complications.^16^, ^17^

Remarkably, GDF15 plays a critical role in the attenuation of hyperglycemia‐induced oxidative stress and inflammation by inhibiting the generation of reactive oxygen species (ROS) and activation of nuclear factor kappa B (NF‐κB), respectively.^6^, ^18^, ^70^ High blood glucose and p53 increase the expression of GDF15 in adipose tissue, thus inhibition of p53 or reduction of blood glucose by antidiabetic agents could inhibit GDF15 expression.^6^, ^19^ Highly expressed GDF15 in obesity and IR induces the release of pro‐inflammatory cytokines, and in turn, these pro‐inflammatory cytokines induce the expression of GDF15.^20^ High GDF15 expression is linked to the development of endothelial dysfunction due to the exaggerated release of pro‐inflammatory cytokines.^20^, ^76^ It has been suggested that administration of GDF15 therapy at the right stage of type 1 diabetes mellitus could delay clinical onset by potentially preserving the remaining β cells. Further, the administration of GDF15 could increase the success of islet replacement therapies for those patients receiving such treatment. Overall, β‐cell protection by GDF15, if demonstrated in humans, could significantly improve T1D patient outcomes. However, there are still significant questions that need to be addressed by thorough fundamental work to understand the precision, efficacy, and long‐term effects GDF15 in humans.^20^

Increased GDF15 in T2DM could be a compensatory mechanism to reduce lipotoxicity and glucotoxicity. In experimental streptozotocin‐induced DM in mice, GDF15 serum level was increased within the first 7 days.^21^, ^22^ A case‐controlled study involving 75 T2DM patients and 29 with impaired fasting glucose compared with 137 healthy control subjects revealed that GDF15 serum levels were higher in patients with T2DM and impaired fasting glucose and correlated with IR, BMI, and age.^23^, ^24^ Kempf et al. investigated baseline GDF15 and its levels over the subsequent 4 years in 496 obese nondiabetic patients. They revealed that GDF15 serum level was linked to IR and abdominal obesity.^25^ Therefore, GDF15 serum level is considered a predictor of the development of IR and impaired fasting glucose in obese nondiabetic patients.^25^, ^26^ Furthermore, GDF15 serum levels have been reported to be augmented in the early manifestations of T2DM and were increased 2‐fold in women with T2DM.^27^, ^28^ This increment in GDF15 serum level was believed to counteract T2DM‐induced inflammatory reactions with anti‐inflammatory effects.^27^ It is interesting to note that GDF15 seems to be a possible biomarker to detect subjects at higher risk for the development of T2DM. A case–control study that included 17 obese nondiabetic women, 14 obese women with T2DM, and 23 healthy lean women revealed that GDF15 levels were elevated in both the obese and T2DM groups compared to controls.^27^ Serum GDF15 positively correlated with body weight, body fat, serum levels of triglycerides, glucose, hemoglobin A1c (HbA1c), and C‐reactive protein, and it was inversely related to serum high‐density lipoprotein cholesterol. Fat mRNA GDF15 expression did not significantly differ between lean and obese women, but it was significantly higher in subcutaneous than in visceral fat in both groups.^27^ Thus, elevated GDF15 levels in patients with obesity are further increased by the presence of T2DM.

Therefore, long‐term prospective and longitudinal studies are warranted to determine the time at which GDF15 levels start increasing in prediabetes patients before the development of T2DM.

### 1.2 GDF15 and metformin

**FIGURE 2:**
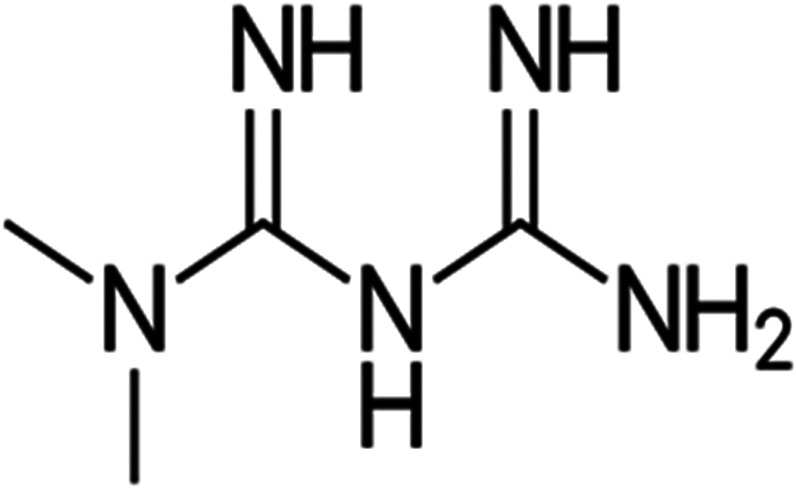
Chemical structure of metformin

Metformin is derived from the natural product galegine of *Galega officinalis*.^29^, ^30^ Galegine was found to reduce blood glucose in humans in 1920, but proved to be very toxic.^29^ Later, two derivatives of galegine, phenformin and metformin, were identified and introduced in the clinical use for the management of T2DM in the 1950s.^29^, ^31^ Phenformin was withdrawn from the market due to toxic adverse effects, leaving only metformin in use.^29^, ^32^ Metformin belongs to the biguanide group containing the guanidine molecule and additional substitutions (Figure 2).

Unlike other synthetic antidiabetic agents, metformin is derived from natural products. Despite the long clinical use of metformin for more than 70 years, its mechanism of action at the molecular level is still debated.^71^ Metformin acts by activating adenosine monophosphate protein kinase (AMPK), which restores and controls energy homeostasis through the activation of the catabolic pathway.^13^, ^33^ Moreover, AMPK is also activated by starvation, mitogen‐activated protein kinase (MAPK), and the mechanistic target of rapamycin (mTOR) pathway in the lysosomal site.^71^ In addition, metformin is accumulated 1000‐fold more within the mitochondria than in the extracellular medium due to its positive charge.^29^, ^34^ It is orally active, and approximately 70% is absorbed from the small intestine. Metformin is not bound to plasma proteins and is not metabolized, so it is excreted unchanged by the kidneys. It is mainly concentrated in the skeletal muscles, pancreas, and adipose tissue.^35^, ^36^ Metformin is mainly used in the management of T2DM and polycystic ovarian syndrome by enhancing insulin sensitivity. Besides, it has pleiotropic effects including antiviral, antibacterial, anticancer, and anti‐inflammatory effects.^37^, ^38^

The mechanism by which metformin reduces body weight is unknown, though independent clinical trials observed that metformin increases the circulating level of GDF15 and inhibits the feeding center inducing satiety.^39^, ^40^ Oral administration of metformin increases GDF15 levels in mice with subsequent attenuation of weight gain in response to a high‐fat diet.^39^, ^41^ Remarkably, GDF15 serum level is regarded as a biomarker of metformin use in T2DM.^10^ A total of 237 biomarkers were assayed in the baseline serum of 8401 participants (2317 receiving metformin) in the Outcome Reduction with Initial Glargine Intervention (ORIGIN) trial, and GDF15 serum levels were found to be strongly linked to metformin use.^10^ Therefore, GDF15 levels are a biomarker for the use of metformin in people with dysglycemia, and its concentration reflects the dose of metformin. In this context, extended‐release metformin may produce a time‐dependent increase in GDF15 serum level. Thus, metformin increases GDF15 serum levels in the dose and time‐dependent effects.^10^

Indeed, induced GDF15 activates a specific central receptor in the brain called glial‐derived neurotrophic factor (GDNF) family receptor α‐like (GFRAL), which is highly expressed in the brain stem to induce taste aversion.^39^, ^42^ Thus, GFRAL antagonists attenuate the weight‐lowering effect of metformin.^39^ Metformin had effects on both energy intake and energy expenditure that were dependent on GDF15, but retained its ability to lower circulating glucose levels in the absence of GDF15 activity. In this state, metformin elevates circulating levels of GDF15, which is necessary to obtain its beneficial effects on energy balance and body weight, major contributors to its action as a chemopreventive agent.^39^ Likewise, the metformin‐mediated effect through GDF15 can control the metabolic effect and energy balance.^42^, ^43^ Moreover, the overexpression of GDF15 promotes the lean phenotype in animals with experimental obesity and T2DM through the activation of the GFRAL receptor.^44^ Yang and his coworkers recently revealed that metformin promotes the expression of GDF15 in the distal small intestine in mice.^45^ This finding suggests that the small intestine could be a potential site for metformin effects.^45^ Of note, an in vitro study revealed that metformin increased expression in human cell lines 26‐fold.^46^ Surprisingly, metformin increased GDF15 in a dose‐dependent manner in a study that involved 8401 patients with dysglycemia.^47^ Similarly, metformin regulates body weight in human immunodeficiency virus (HIV)‐infected patients through GDF15.^47^ Moreover, metformin reduces blood glucose through the modulation of bacterial flora and increases the release of glucagon‐like peptides.^47^, ^48^

**FIGURE 3:**
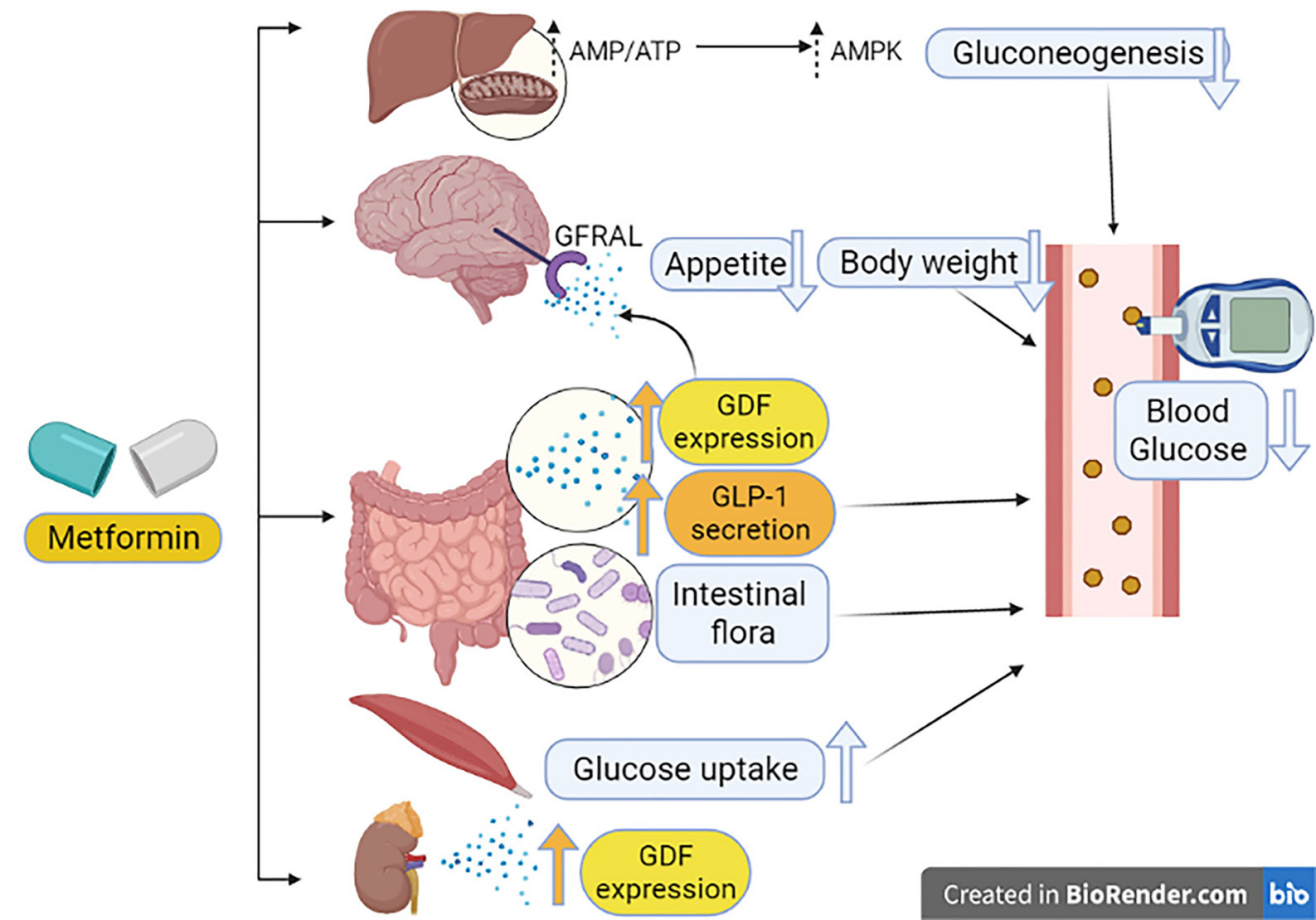
Mechanism of weight reduction by metformin: Metformin reduces blood glucose in addition to its activation of activated monophosphate protein kinase (AMPK), it also acts through the modulation of bacterial flora and increasing the release of glucagon‐like peptide. As well, metformin induces the expression of growth differentiation factor 15 (GDF15) in small intestine and kidney that activates glial‐derived neurotrophic factor (GDNF) family receptor α‐like (GFRAL) in the brain causing the reduction of appetite with subsequent weight reduction

These observations suggest that metformin therapy in T2DM patients may reduce body weight through GDF15, which is independent of the improvement of insulin sensitivity (Figure 3).

## 2 GDF15 AND DIABETIC COMPLICATIONS

### 2.1 Thrombosis

GDF15 is regarded as a prognostic biomarker of pulmonary embolism in patients with cardiovascular diseases.^49^ A prospective cohort study involving 123 patients with acute pulmonary embolism revealed that GDF15 serum level was higher and correlated with 30‐day mortality.^49^ In addition, there is evidence proposed that GDF15 serum level appears to be linked with stroke in patients with atrial fibrillation.^50^ An observational study including 894 patients with atrial fibrillation with or without left atrial thrombus revealed that GDF15 serum level was higher in patients with atrial thrombus compared to patients with atrial fibrillation without atrial thrombus.^50^ Inflammatory reactions induce thrombosis and release of GDF15 from activated macrophages.^51^ However, GDF15 knockout mice experience accelerated thrombosis compared to wild‐type mice.^52^ Moreover, in vitro study demonstrated that GDF15 could inhibit platelet aggregation.^52^ Thus, GDF15 might not be the putative cause of thrombosis but a compensatory mechanism against the development of thromboembolic disorders in various cardiovascular complications.^51^

On the other hand, T2DM patients are at higher risk for the development of pulmonary hypertension and pulmonary embolism independent of smoking, hypertension, heart failure, and coronary artery disease.^53^, ^77^ The pathogenesis of this link has not yet been identified, although endothelial dysfunction, oxidative stress, and hypercoagulability in T2DM could be the association link.^53^, ^54^ Thrombotic events are augmented in T2DM patients due to platelet hyperreactivity, endothelial dysfunction, and uncontrolled activation of the coagulation pathway.^55^ IR, low‐grade inflammation, and oxidative stress might be the potential causes of the hypercoagulant state in T2DM.^55^

Of note, oxidative stress and inflammation interact in the development of diabetic atherosclerosis. Intracellular hyperglycemia promotes the production of mitochondrial ROS, increases formation of intracellular advanced glycation end products, activation of protein kinase C, and increases polyol pathway flux. ROS directly increase the expression of inflammatory and adhesion factors, formation of oxidized low‐density lipoprotein, and IR. They activate the ubiquitin pathway, inhibit the activation of AMPK and adiponectin, and decrease endothelial nitric oxide synthase activity, all of which accelerate atherosclerosis in T2DM.^72^ It has been shown that GDF15 is regarded as a strong and independent predictor of mortality and disease progression in patients with atherosclerosis and coronary artery disease.^72^ Elevated GDF15 has been shown to promote inflammation and angiogenesis, implying that GDF15 may play an important role in the pathogenesis of atherosclerosis.^73^ While GDF15 is a cardiovascular risk factor, whether GDF15 directly contributes to the development of atherosclerosis has not been established, and the precise relationships between GDF15 and atherosclerosis are not fully understood. GDF15 was originally identified as a factor overexpressed in activated macrophages to regulate inflammation, which is involved in all stages of atherosclerosis, from its initiation and progression to its thrombotic complications.^74^ These observations suggest increasing GDF15 in T2DM is associated with the risk for the development of atherosclerosis and thrombosis.

Therefore, thrombosis in T2DM could be due to elevation of GDF15, which is associated with the risk of thrombosis. Thus, targeting oxidative stress and immunoinflammatory changes may reduce the risk of thrombosis and pulmonary embolism regardless of GDF15.^49^, ^50^ In this context, higher GDF15 in T2DM patients with atherosclerosis and thrombotic complications could be a compensatory mechanism against reverse oxidative and inflammatory‐induced thrombotic disorders.

## 3 DIABETIC NEPHROPATHY

Diabetic nephropathy (DN) is the most common cause of the development of end‐stage kidney disease in the diabetic population characterized by microalbuminuria.^56^ GDF15 is associated with the development of DN as it is regarded as an independent predictor for the development of DN in diabetic patients.^57^ A prospective observational study involving 451 diabetic patients with DN compared to 440 diabetic patients without DN revealed that elevation of GDF15 was correlated with the decline in glomerular filtration rate and deterioration of kidney function.^57^, ^78^ A recent study conducted by Perez‐Gomez et al.^58^ confirmed that urinary GDF15 level is regarded as a biomarker for the development of chronic kidney disease (CKD). Thus, urinary GDF15 level is correlated with mortality and an abnormal pattern of kidney architecture in CKD patients.^58^ Notably, the GDF15 level might be used in the diagnosis and evaluation of DN in T2DM patients.^59^ An observational study comprising 30 T2DM patients, 10 with macroalbuminuria and 20 with microalbuminuria, showed that the GDF15 level was higher in T2DM patients with macroalbuminuria compared to those with microalbuminuria.^59^

These findings suggest that GDF15 could be a predictive biomarker for the development of DN in T2DM patients.

## 4 DIABETIC NEUROPATHY

Diabetic neuropathy is one of the most common adverse comorbidities in T2DM patients, leading to distal symmetrical polyneuropathy characterized by sensory disturbances and autonomic dysfunction.^60^ In advanced diabetic neuropathy, Charcot osteoarthropathy and diabetic pain are developed and associated with limited quality of life.^60^ It has been shown that higher circulating levels of GDF15 are associated with progression of diabetic neuropathy.^61^ A comparative study including 241 diabetic patients and 42 nondiabetic patients illustrated that GDF15 level was correlated with the amplitude and latency of motor and sensory nerves.^61^ Thus, the GDF15 level is regarded as an independent risk factor for the development of diabetic neuropathy. Different experimental studies revealed that GDF15 knockout mice had a greater risk for neuron loss, and exogenous administration of GDF15 improved the survival of dopaminergic neurons.^62^, ^63^ Higher GDF15 levels in diabetic neuropathy mirror inflammatory and oxidative stress disorders, which are implicated in the pathogenesis of microvascular dysfunction and progression of diabetic neuropathy.^61^ Thus, the association between GDF15 and diabetic neuropathy could be a compensatory mechanism to counter‐regulate the development of microvascular dysfunction.

## 5 DIABETIC RETINOPATHY

Diabetic retinopathy (DR) has been declining since 1980 with the improvement of diabetes control. The prevalence of DR and linked visual impairment increased between 1990–2015 due to an increased prevalence of T2DM.^64^ A systematic review revealed that the incidence of DR was 2.2%–12.7%, mainly in individuals with mild disease.^65^ Remarkably, different studies demonstrated that GDF15 level is correlated with the progression of DR. For example, a study by Chung and colleagues revealed a strong relationship between GDF15 and DR in T2DM patients.^66^ A cross‐sectional study that including 235 T2DM patients with and without DR illustrated that the GDF15 level was higher and correlated with the severity of DR compared with T2DM patients without DR (*p* = 0.03, CI = 1.05–3.03).^66^ Ilhan et al.^67^ disclosed that the GDF15 level was correlated with the severity of retinal inflammation and vitroretinal disorders. An observational follow‐up study showed that GDF15 levels were correlated with mortality and diabetic complications including DR in T2DM patients.^68^ In addition, the GDF15 level could be a prognostic biomarker predicting the progression of DR.^69^

These findings propose that GDF15 might be a diagnostic and prognostic biomarker that reflects the development of DR in T2DM patients.

Taken together, the GDF15 level is linked and correlated with the progression of diabetic complications including thrombosis, DN, diabetic neuropathy, and DR. However, this association needs to be verified in different preclinical and clinical studies.

## 6 CONCLUSIONS

GDF15 acts through the activation of GFRAL receptors in the brain, which are responsible for taste aversion. GDF15 has anti‐inflammatory effects, so its increase in T2DM and other cardiometabolic disorders counterbalances the inflammatory milieu. Metformin, the first‐line therapy in the management of T2DM, induces the expression of GDF15, reducing appetite with the induction of weight loss in both diabetic and nondiabetic patients. Metformin therapy in T2DM patients may reduce body weight through GDF15, which is independent of the improvement of insulin sensitivity. In addition, the GDF15 level is linked and correlated with the progression of diabetic complications including thrombosis, DN, diabetic neuropathy, and DR. However, this association needs to be verified in different preclinical and clinical studies. The present review cannot give a final conclusion in this regard. Therefore, experimental, preclinical, and clinical studies are warranted to confirm the potential role of GDF15 in T2DM patients.

## AUTHOR CONTRIBUTIONS

**Hayder M. Al‐kuraishy and Ali I. Al‐Gareeb:** Conceptualization; writing of the review. **Athanasios Alexiou, Marios Papadakis, Hebatallah M. Saad, and Gaber El‐Saber Batiha:** Preparation of figures; writing, correcting, and amending of the article. **Eman Hassan Nadwa, Sarah M. Albogami, and Mohammed Alorabi:** editing and polishing of the manuscript and responding to reviewers' comments. All authors contributed significantly to the manuscript and approved the submitted version.

## FUNDING INFORMATION

Open Access funding enabled and organized by Projekt DEAL. This work was supported by the University of Witten‐Herdecke Germany.

## DISCLOSURE

None.
